# Supplementary material for: Effect of physician characteristics and knowledge on the quality of dyslipidemia management and LDL–C target goal achievement in China: Subgroup analysis of the Dyslipidemia International Study
Source: J Glob Health. 2017 Sep 26;7(2):020702. doi: 10.7189/jogh.07.020702 (PMC5644362; doi:10.7189/jogh.07.020702)
Supplement: Online Supplementary Document [file jogh-07-020702-s001.pdf]

Electronic supplementary material:
The online version of this article contains supplementary material.

journal of global health

PAPERS

Effect of physician characteristics and knowledge on the quality of dyslipidemia management and LDL–C target goal achievement in China: Subgroup analysis of the Dyslipidemia International Study

Rongjing Ding[1], Ping Ye[2], Shuiping Zhao[3], Dong Zhao[4], Xiaowei Yan[5], Yugang Dong[6], Jihu Li[7], Yuqin Ran[8], Dayi Hu[1]; on behalf of the DYSIS-China Study Investigators

[1] Department of Cardiology, Peking University People's Hospital, Beijing, China
[2] Department of Gerontology, Chinese PLA General Hospital, Medical School of Chinese PLA, Beijing, China
[3] Department of Cardiology, The Second Xiangya Hospital of Central South University, Changsha, China
[4] Institute of Epidemiology, Beijing Anzhen Hospital, Capital Medical University, Beijing, China
[5] Department of Cardiology, Peking Union Medical College Hospital, Beijing, China
[6] Department of Cardiology, The First Affiliated Hospital, Sun Yat-sen University, Guangzhou, China
[7] Outcome Research, Merck Sharp & Dohme (China) Holding Ltd., Shanghai, China
[8] Medical Affairs, Merck Sharp & Dohme (China) Holding Ltd., Shanghai, China

Correspondence to:
Dayi Hu
Department of Cardiology
Peking University People's Hospital
No. 11 Xizhimen South Stree
Xicheng District, Beijing, 100044
China
hudayi080615@163.com

Objective This study aimed to investigate the effect of physicians' characteristics and knowledge of LDL–C target goals on the quality of lipid management in China.

Methods A total of 25 317 dyslipidemia patients who had taken lipid–lowering medication for >3 months were enrolled in our study. Patients' demographic data, medical history, lipid profile, their physician's specialty and professional title and their hospital level as well as their LDL–C goal opinions were recorded.

Results Questionnaires were completed by 926 physicians with 6 different specialties and 4 professional statuses, in 3 different–level hospitals. Most (74.5%) of the physicians recognized the importance of considering LDL–C serum concentration for treating dyslipidemia, and set target LDL–C goals according to the 2007 Chinese guidelines for 83.4% of their patients. The LDL–C goal achievement rate was significantly higher for patients whose physicians' knowledge of LDL–C target goals was consistent with guideline recommendations, compared with those whose physicians' knowledge was inconsistent with the guidelines (60.4% vs 31.1%, $P<0.0001$). Physicians working in tier 1 (odds ration (OR) = 2.95; 95% CI 2.37–3.67), (OR = 1.56; 95% CI 1.34–1.81) and tier 2 (OR = 2.53; 95% CI 2.22–2.88), (OR = 1.16; 95% CI 1.06–1.27) hospitals, specialized in neurology (OR = 1.13; 95% CI 0.93–1.36), (OR = 1.57; 95% CI 1.40–1.77), internal medicine (OR = 1.07; 95% CI 0.90–1.27), (OR = 1.58; 95% CI 1.39–1.80), endocrinology (OR = 1.02; 95% CI 0.87–1.21), (OR = 1.63; 95% CI 1.47–1.82) and being a resident vs attending physician (OR = 1.05; 95% CI 0.92–1.20), (OR = 1.00; 95% CI 1.00–1.19) were independent risk factors for low knowledge of LDL–C target goals and low LDL–C goal achievement.

Conclusion Chinese physicians' characteristics and knowledge of LDL–C target goals were associated with patients' LDL–C goal achievement.

An elevated level of serum cholesterol has been suggested to be the most important risk factor for ischemic cardiovascular disease (CVD) [1–7]. The Cholesterol Treatment Trialists' meta–analysis of statin clinical trials found that a 1 mmol/L reduction in LDL–C reduced major adverse CVD

events by approximately 20% [8,9]. Hence, prescription statin and LDL–C target goal achievement for patients with high– and very high–risk CVD is considered as an important strategy in CVD prevention and the epidemiology of LDL–C target goal achievement has been widely investigated. In 2014, the Dyslipidemia International Study in China (DYSIS–China) revealed that, after almost 10 years of cholesterol education for the primary and secondary prevention of CVD, 45% of high–risk and 60% of very high–risk dyslipidemia patients in China had not attained their LDL–C target goals [10]. Although these data reflect some improvement compared to previous reports that 69% of high–risk and 78% of very high–risk patients in China failed to attain their LDL–C goal between 2004 and 2006 [11], large gaps still exist, when compared with developed countries, regarding LDL–C goal achievement [12]. High–quality care ultimately comes from high–quality health professionals [13]. Previous studies have found that physicians play an important role in lipid management [14–17]. While physicians' attitude and behavior may affect the efficiency of lipid management, inadequate knowledge of LDL–C targets by physicians is also an important factor in dyslipidemia patients' failure to attain their LDL–C goals [6,18]. A previous study has reported that physicians' knowledge of lipid management was related to their professional status [19]. In China, the main characteristics of physicians include their professional status, as well as their specialty, and the quality and region of the hospital where they work. However, it is unclear how Chinese physicians' knowledge of guideline–recommended LDL–C targets and other characteristics affect their patients' LDL–C goal achievement. Therefore, we performed a subgroup analysis of DYSIS–China patients to evaluate the association between Chinese physicians' fundamental knowledge of lipid management and their patients' LDL–C goal achievement rate, and how it relates to the physicians' specialty and professional status, as well as their hospital's quality and location.

METHODS

Patients and study design

The DYSIS–China trial was an observational, cross–sectional, multicenter international study. Participants were recruited at 122 hospitals, including 58 tier 3 (teaching hospital), 31 tier 2 (territory hospital), and 33 tier 1 (community hospital) institutions in 6 representative regions of China between April 2012 and October 2012 (Table S1 in **Online Supplementary Document**). Patients who were 45 years of age or older and had been treated with a lipid–lowering drug for at least 3 months were included in our study. Data for each patient were collected from the baseline clinical examination, medical records, and a single outpatient follow–up visit. Lipid–lowering medications included statins, cholesterol absorption inhibitors, fibrates, nicotinic acid, and Xuezhikang. The 10–year risk of CVD (10YRCVD) for each patient was classified as follows, based on the 2007 Chinese Guidelines on Prevention and Treatment of Dyslipidemia in Adults: (a) very high–risk (CVD with diabetes or acute coronary syndrome); (b) high–risk (coronary heart disease with a 10YRCVD of 10% to 15%; (c) moderate risk (10YRCVD of 5% to 10%); and (d) low–risk (10YRCVD of <5%).

All of the participating physicians were asked to complete a questionnaire that asked whether they regarded guideline recommendations for LDL–C as an important clinical reference for lipid management. If the physician answered yes, he/she was asked to select an LDL–C target goal based on the patient's relevant risk category, which was defined in the 2007 Chinese lipid management guidelines as follows: (a) <4.14 mmol/L (<160 mg/dL) for low–risk patients; (b) <3.37 mmol/L (<130mg/dL) for moderate–risk patients; (c) <2.59 mmol/L (<100 mg/dL) for high–risk patients; (d) <2.07 mmol/L (<80 mg/dL) for very high–risk patients; (e) other; and (f) not sure. Consistency between each physician–suggested LDL–C target goal and the LDL level recommended by the 2007 guidelines was defined as follows: (a) Yes (consistent), if the physician–suggested LDL–C target goal was lower than or equal to the LDL–C level recommended by the guidelines. (b) No (not consistent), if the physician's suggested LDL–C target goal was higher than that recommended by the guidelines. Data for each physician category including professional status (professor, associate professor, attending physician, or resident physician), specialty (including internal medicine, general medicine, cardiology, neurology, endocrinology, and geriatrics), as well as the level of their hospital (tier 1, tier 2, or tier 3), were collected. Each patient provided written informed consent before participation, and our study protocol was approved by the ethics committee of each participating hospital. All of the hospitals and physicians consented to the publication of information related to hospital status and the treating physicians' specialties and professional titles.

Statistical analysis

All of the statistical analyses were performed using the SAS, version 9.1, software (SAS Institute, Cary, NC, USA). A hypothesis test could not be used for the principal analysis, due to the primarily descriptive nature of our study design. Categorical variables are presented as absolute numbers and percentages. Continuous variables are reported as the mean ± standard deviation (SD). Intergroup differences in categorical variables were evaluated using chi–square analysis or Fisher exact test, depending on the number of patients in each group. Intergroup differences in continuous variables were evaluated using an analysis of variance. Logistic regression models were used to calculate the odds ratio and 95% confidence interval for the probability of consistency between the physician–suggested and guideline–recommended LDL–C target levels based on hospital status, type of physician or department, and adjusting for the 10YRCVD category, comorbidities, or other risk factors, including hypertension, body mass index (BMI) ≥28 kg/m^2, male >45 years or female >55 years, smoking status, family history of premature CVD, and hospital level, as well as the physician's specialty and professional title. All of the evaluations were 2–tailed, and results with $P<0.05$ were considered to be statistically significant.

RESULTS

Baseline patient characteristics

A total of 25 317 dyslipidemia patients (51.3% male) with a mean age of 65.4 years were included in our study. Most of the patients (97.5%) were Han Chinese. The most prevalent comorbidities were hypertension (65.8%), diabetes mellitus (34.8%), and coronary heart disease (37.2%). Behavioral risk factors included sedentary lifestyle (19.7%) and smoking (12.4%). The distribution of patients based on 10YRCVD was 12.2% for the very high–risk, 58.9% for high–risk, 11.0% for moderate–risk, and 17.9% for the low–risk groups. The normal dosage of statin is equivalent to simvastatin 20–40 mg/d (71%) (**Table 1**).The LDL–C goal achievement rates for each category are listed in **Table 1**.

Physician characteristics

A total of 926 physicians participated in our study, 168 (18.1%) of whom were from tier 1 hospitals, 199 (21.5%) from tier 2 hospitals, and 559 (60.4%) from tier 3 hospitals. The physicians' specialties were as follows: 228 (24.6%) cardiology, 156 (16.3%) neurology, 185 (19.3%) endocrinology, 113 (12.2%) geriatrics, 210 (22.7%) internal medicine, and 34 (3.7%) general medicine. At tier 1 hospitals, all of the physicians were from the internal medicine department, 73.8% of whom were attending physicians and residents. Most physicians at tier 2 hospitals were associate professors or attending physicians from the cardiology or internal medicine departments, followed by the endocrinology, neurology, and geriatric departments. In tier 3 hospitals, more physicians were professors in all departments, compared with tier 2 and tier 1 hospitals (24.7% vs 11.1% $P<0.0001$ and 24.7 vs 6.5% $P<0.0001$, respectively). Also the distribution of physicians' status differed between some hospital departments particularly within the Tier 2 and tier 3 groups (**Table 2**).

Physician characteristics and knowledge of LDL–C targets as a clinical reference for LDL–C goal achievement

The proportion of physicians who considered the guideline–recommended LDL–C goal as an important clinical reference was different depending on physician characteristics, and was higher at tier 2 and tier 3 hospitals. It was also somewhat higher among physicians who specialized in cardiology, neurology, and geriatrics. Physicians' low knowledge of LDL–C goal as a clinical reference predicted lower LDL–C goal achievement. At tier1, tier 2 and tier 3 hospitals, 65.4%, 81.8% and 75.2% of the physicians recognized that LDL–C goal is important in clinical practice, the overall goal achievement rates were only 47.7%, 58.0% and 57.6%, respectively (**Figure 1A**, Table S2 in **Online Supplementary Document**), and the lowest rate of LDL target goal attainment was in tier 1 hospitals. Regarding different specialties, 73.5% to 78.4% of the physicians recognized that LDL–C goal achievement is important in clinical practice, and the goal achievement rate ranged from 41.5% to 66.0% across the various specialties. The greatest difference between patients' goal attainment and physician perception was found to be among endocrinologists (**Figure 1B**, Table S2 in **Online Supplementary Document**). The lowest LDL–C goal achievement rate and the least knowledge by physicians regarding LDL–C as an important clinical reference existed in the Northeast (**Figure 1C**, Table S2 in **Online Supplementary Document**), and among resident physicians (**Figure 1D**, Table S2 in **Online Supplementary Document**).

PAPERS

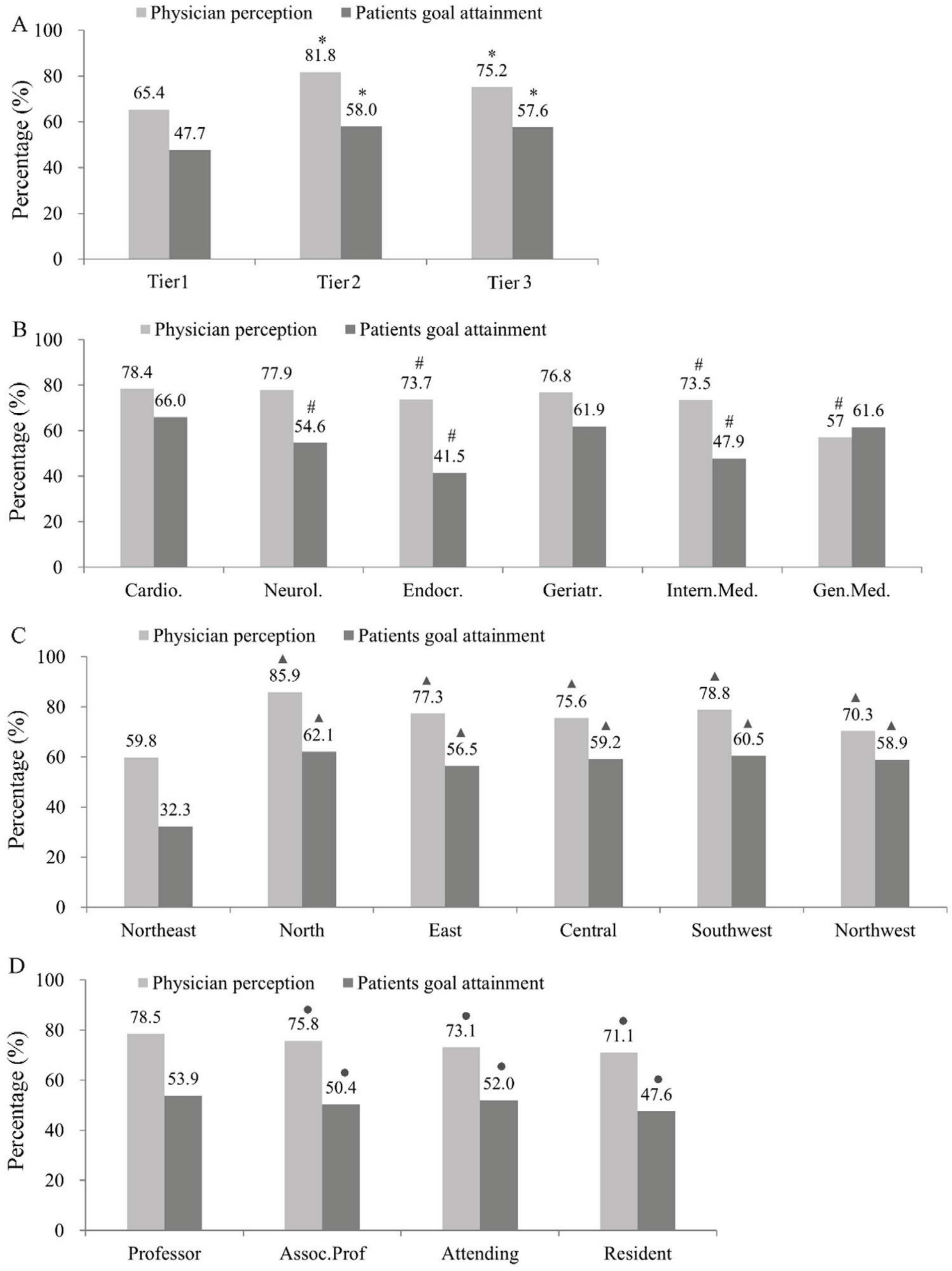

Figure 1. Comparison of questionnaire results regarding whether physicians recognized that the guideline–recommended low–density lipoprotein cholesterol (LDL–C) goal is an important clinical reference for dyslipidemia management and the LDL–C goal achievement rates based on (**A**) hospital level, (**B**) physician specialty, (**C**) professional status, and (**D**) physician perception. *P<0.05, compared to Tier1, #P<0.05, compared to Cardiology department, ▲P<0.05, compared to Northeast, ●P<0.05, compared to Professor title.

Effects of physician characteristics and knowledge of guideline–recommended LDL–C targets on LDL–C goal achievement

Our study showed that less than 75% of physicians in China are familiar with guideline–recommended LDL–C targets, regardless of physician characteristics. There was an association between the rate of LDL–C goal achievement by patients and their physicians' knowledge of LDL–C targets. The concordance between physicians' knowledge of LDL–C targets and guideline recommendations was the lowest in tier 1 hospitals (Figure 2A, Table S3 in **Online Supplementary Document**), endocrinology departments. (Figure 2B, Table S3 in **Online Supplementary Document**), Northeast China (Figure 2C, Table S3in **Online Supplementary Document**) and among resident physicians (Figure 2D, Table S3 in **Online Supplementary Document**).

Table 1. Patients' basic characteristics and LDL–C goal achievement rate

	Number (%) N = 25 317	LDL–C goal achievement n (%)
**Age category:**		
≥65 years	13 100 (51.7%)	7803 (59.6%)
**Gender:**		
Male	12 975 (51.3%)	8431 (65.0%)
Female	12 342 (48.7%)	7140 (57.9%)
**Coronary heart disease**	9420 (37.2%)	5247 (55.7%)
**Cerebrovascular disease**	4281 (16.9%)	2304 (53.8%)
**Peripheral arterial disease**	263 (1.0%)	153 (58.2%)
**Chronic kidney disease**	1846 (7.3%)	1095 (59.3%)
**Diabetes mellitus**	8798 (34.8%)	3978 (45.3%)
**Hypertension**	16 650 (65.8%)	10075 (60.5%)
**Other cardiovascular risk factors:**		
Smoking:		
Current	3143 (12.4%)	1902 (60.5%)
Quit	4445 (17.6%)	2881 (64.8%)
Never	17 729 (70.0%)	10788 (60.9%)
HDL–C <1.04mmol/L	6682 (26.4%)	4468 (68.9%)
Family history of CHD	2294 (9.1%)	1381 (60.2%)
BMI >28kg/m^2	3457 (13.7%)	1947 (56.3%)
Family history of early onset of ischemic cardiovascular disease	2294 (9.1%)	1381 (60.2%)
Sedentary lifestyle	4997 (19.7%)	2962 (59.3%)
**10YRCVD category:**		
Very high	3092 (12.2%)	1226 (40.0%)
High	14 916 (58.9%)	8174 (54.8%)
Moderate	2782 (11.0%)	2041 (73.4%)
Low	4527 (17.9%)	4130 (91.2%)
**Statin dosage potency:***		
Potency 1	299 (1.2%)	192/299 (64.2%)
Potency 2	2605 (10.3%)	1512/2605 (58.0%)
Potency 3	9958 (39.3%)	6014/9958 (60.4%)
Potency 4	7179 (28.4%)	4547/7179 (63.3%)
Potency 5	1725 (2.9%)	1083/1725 (62.8%)
Potency 6	96	57/96 (59.4%)

HDL–C – high density lipoprotein cholesterol, CHD – chronic disease, 10YRCVD – 10–y risk of cardiovascular disease

*Statin dosage level: Potency1 is equivalent to simvastatin 5 mg/d; Potency 2 is equivalent to simvastatin 10 mg/d; Potency 3 is equivalent to simvastatin 20 mg/ day; Potency 4 is equivalent to simvastatin 40 mg/d; Potency is equivalent to simvastatin 80 mg/d; Potency 6 is equivalent to Atorvastatin 80 mg/d.

Effects of physicians' knowledge of LDL–C as a clinical reference and guideline–recommended LDL–C targets on the rate of LDL–C goal achievement

In total, 74.5% of physicians recognized that LDL–C is an important clinical reference. The LDL–C goal achievement rate was significantly higher among those patients whose physicians' perceptions of the LDL–C target goal were consistent with guideline recommendations (60.4% vs 31.1%, $P<0.0001$, **Table 3**). The LDL–C goal achievement rates in patients with very high, high, and moderate risk whose physicians' knowledge of the LDL–C target goal was consistent with guideline recommendations were 39.9%, 51.5%, and 70%, respectively. The corresponding rates among patients whose physicians' knowledge of the LDL–C target goal was inconsistent with guideline recommendations were 29.7%, 32.1%, and 30.1%, respectively ($P<0.0001$ for all). These results suggest that the LDL–C goal achievement rate significantly correlated with physicians' knowledge of the guideline–recommended LDL–C target goal.

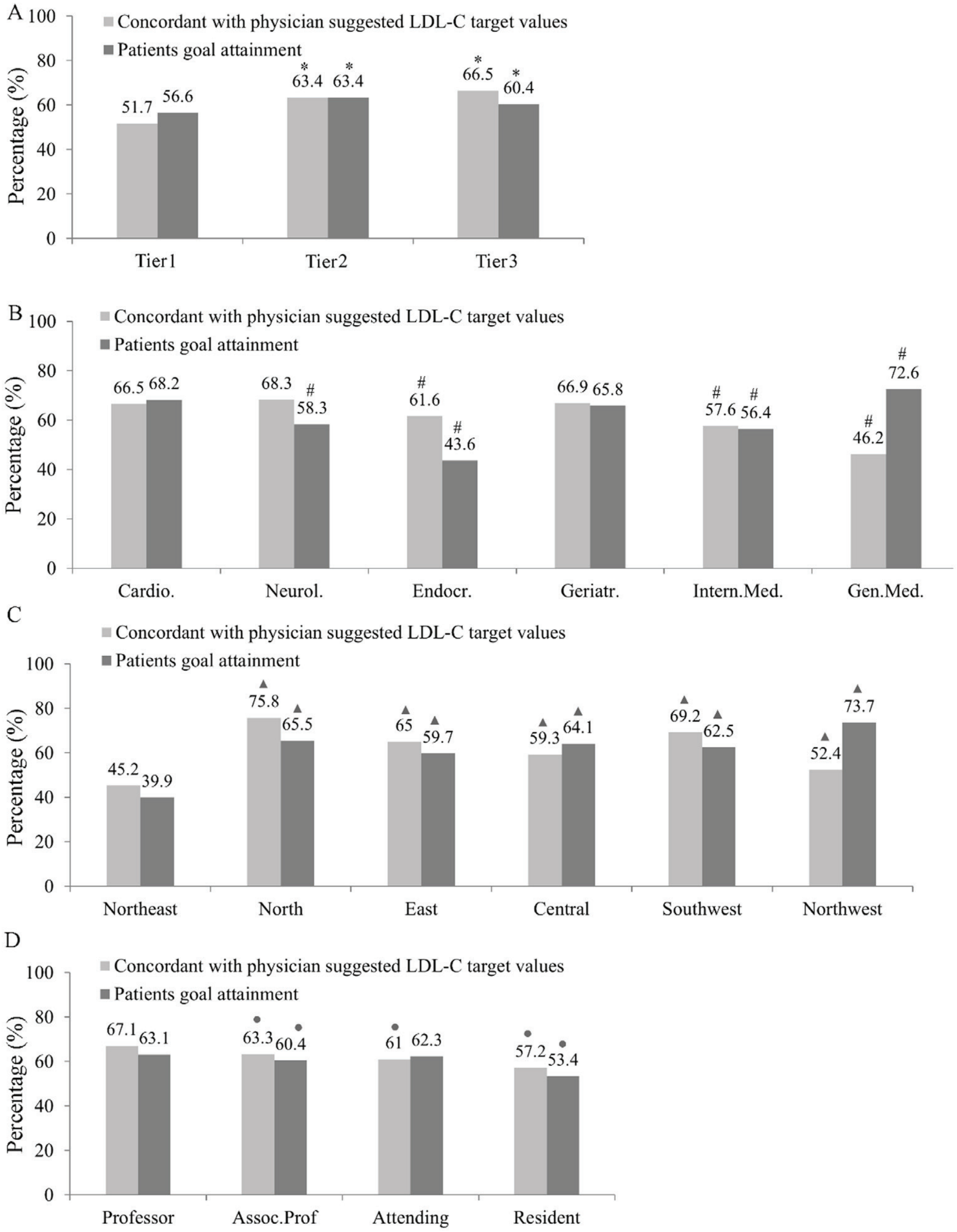

Figure 2. LDL–C goal achievement rate and consistency between physicians' knowledge of LDL–C target goal and guideline recommendations based on (**A**) hospital level, (**B**) physician specialty, (**C**) China's geographic regions, and (**D**) professional status. *P<0.05, compared to Tier 1, #P<0.05, compared to Cardiology department, ▲P<0.05, compared to Northeast, ●P<0.05, compared to Professor title.

Predictors of inconsistent physicians' knowledge with guideline–recommended LDL–C target goals using multivariate logistic regression analysis

Multivariate logistic regression analysis revealed that predictors of inconsistency between physicians' perceptions of the LDL–C target goal and 2007 Chinese guideline recommendations included patients diagnosed with diabetes, coronary artery disease, cerebrovascular disease, and peripheral arterial disease. Other predictors of inconsistency between physicians' perceptions of the LDL–C target goal and 2007 Chinese guideline recommendations included the following physician characteristics: working in a tier 1 or tier 2 hospital, specializing in neurology, endocrinology, geriatrics, or general medicine, and being a resident physician. In addition, there were differences regarding the regions (Table 4).

Table 2. Characteristics of physicians based on hospital status and specialty

Hospital status		Number of physicians (%)						
		Cardiology	Neurology	Endocrinology	Geriatrics	Internal Medicine	General Medicine	Total*
Tier 1	Professor				0	10 (7.5%)	1 (2.9%)	11 (6.5%)[a]
	Assoc.Prof.				0	26 (19.4%)	7 (20.6%)	33 (19.6%)[a]
	Attending				0	50 (37.3%)	17 (50.0%)	67 (39.9%)[a]
	Resident				0	48 (35.8%)	9 (26.5%)	57 (33.9%)[a]
	Total				0	134 (100.0%)	34 (100.0%)	168 (100.0%)
Tier 2	Professor	7 (9.3%)	4 (36.4%)	4 (11.4%)	0	7 (9.2%)		22 (11.1%)[a]
	Assoc.Prof.	19 (25.3%)	3 (27.3%)	11 (31.4%)	2 (100.0%)	22 (28.9%)		57 (28.6%)[b]
	Attending	30 (40.0%)	1 (9.1%)	10 (28.6%)	0	30 (39.5%)		71 (35.7%)[a]
	Resident	19 (25.3%)	3 (27.3%)	10 (28.6%)	0	17 (22.4%)		49 (24.6%)[b]
	Total	75 (100.0%)	11 (100.0%)	35 (100.0%)	2 (100.0%)	76 (100.0%)		199 (100.0%)
Tier 3	Professor	41 (26.8%)	34 (23.4%)	28 (18.7%)	35 (31.5%)			138 (24.7%)[b]
	Assoc.Prof.	47 (30.7%)	47 (32.4%)	42 (28.0%)	24 (21.6%)			160 (28.6%)[b]
	Attending	46 (30.1%)	45 (31.0%)	50 (33.3%)	41 (36.9%)			182 (32.6%)[a]
	Resident	19 (12.4%)	19 (13.1%)	30 (20.0%)	11 (9.9%)			79 (14.1%)[c]
	Total	153 (100.0%)	145 (100.0%)	150 (100.0%)	111 (100.0%)			559 (100.0%)
Total	Professor	48 (21.1%)	38 (24.4%)	32 (17.3%)	35 (31.0%)	17 (8.1%)	1 (2.9%)	171 (18.5%)
	Assoc.Prof.	66 (28.9%)	50 (32.1%)	53 (28.6%)	26 (23.0%)	48 (22.9%)	7 (20.6%)	250 (27.0%)
	Attending	76 (33.3%)	46 (29.5%)	60 (32.4%)	41 (36.3%)	80 (38.1%)	17 (50.0%)	320 (34.6%)
	Resident	38 (16.7%)	22 (14.1%)	40 (21.6%)	11 (9.7%)	65 (31.0%)	9 (26.5%)	185 (20.0%)
	Total	228 (100.0%)	156 (100.0%)	185 (100.0%)	113 (100.0%)	210 (100.0%)	34 (100.0%)	926 (100.0%)

Assoc. Prof – Associate Professor

*Indicates containing sub–groups which are marked with a, b and c. Different letter marks indicate significant differences of the indicated professional title group within departments of the hospitals, same letters indicate no significant differences ($P>0.05$).

Table 3. LDL–C goal achievement rates according to physicians' knowledge of LDL–C as a clinical reference and consistency between physicians' acceptance of LDL–C target goal and guideline recommendations based on 10YRCVD*

		LDL–C goal achievement (yes/total) for 10YRCVD categories				
		Very high	High	Moderate	Low	Total
Total		1226/3092 (39.7%)	8174/14 916 (54.8%)	2041/2782 (73.4%)	4130/4527 (91.2%)	15 571/25 317 (61.5%)
LDL–C as a clinical reference	Yes (74.5%)	836/2363 (35.4%)	5411/11 222 (48.2%)	1393/2054 (67.8%)	2852/3231 (88.3%)	10 492/18 870 (55.6%)
	No	164 (29.8%)	780 (27.4%)	138 (27.3%)	305 (34.0%)	1387 (28.9%)
	P–value	NA	NA	NA	NA	0.0014
Consistency with guideline–recommended LDL–C target goal‡	Yes (83.4%)	515/1290 (39.9%)	4777/9270 (51.5%)	1358/1940 (70%)	2852/3231 (100%)	9502/15 731 (60.4%)
	No†	318/1069 (29.7%)	624/1941 (32.1%)	34/113 (30.1%)	NA	976/3123 (31.3%)
	P–value	$P<0.0001$	$P<0.0001$	$P<0.0001$	NA	$P<0.0001$

HDL–C – high density lipoprotein cholesterol, CHD – chronic disease, 10YRCVD – 10–year risk of cardiovascular disease

*10YRCVD categories were based on serum LDL–C levels of <2.07 mmol/L (<80 mg/dL) for very high risk, <2.59 mmol/L (<100 mg/dL) for high risk, <3.37 mmol/L (<130 mg/dl) for moderate risk, and <4.14 mmol/L (<160 mg/dL) for low risk.

†Included patients in whom the risk classification was lower than guideline recommendations.

‡2007 Chinese lipid management guidelines as the reference.

Predictors of failure for not achieving guideline–recommended LDL–C target goals using multivariate logistic regression analysis

The result showed that predictors of failure to achieve the LDL–C goal included patients with diabetes, coronary artery disease, cerebrovascular disease, BMI>28 kg/m^2 and male ≥45 years or female ≥55 years, physician working in a tier 1 or tier 2 hospital, specializing in neurology, endocrinology or general medicine, and being a resident physician (**Table 5**).

PAPERS

Table 4. Multivariate logistic regression analysis of consistence between physician suggested–LDL–C target goal and recommendations of 2007 Chinese guidelines

	Parameter estimation value	OR	95% CI	P–value
**Comorbidity (yes vs no):**				
Diabetes mellitus	1.24	3.47	3.15–3.83	<0.0001
Coronary heart disease	1.33	3.78	3.42–4.18	<0.0001
Cerebrovascular disease	0.41	1.51	1.33–1.70	<0.0001
Peripheral arterial disease	1.08	2.94	2.13–4.06	<0.0001
Chronic kidney disease	–0.07	0.93	0.80–1.09	0.3755
**Risk factors:**				
Hypertension (yes vs no)	0.04	1.05	0.94–1.16	0.4118
BMI >28kg/m^2	–0.01	0.99	0.87–1.13	0.9256
Male ≥45 years or Female ≥55 years (yes vs no)	0.22	1.24	1.03–1.50	0.0227
Smoking (yes vs no)	–0.11	0.89	0.77–1.03	0.1196
Family history of early onset of ischemic cardiovascular disease (yes vs no)	–0.13	0.88	0.75–1.03	0.1198
**Hospital level:**				
Tier 1 vs Tier 3	1.08	2.95	2.37–3.67	<0.0001
Tier 2 vs Tier 3	0.93	2.53	2.22–2.88	<0.0001
**Specialty:**				
Neurology vs Cardiology	0.12	1.13	0.93–1.36	0.2180
Endocrinology vs Cardiology	0.02	1.02	0.87–1.21	0.7733
Geriatrics vs Cardiology	0.24	1.27	1.06–1.53	0.0112
Internal medicine vs Cardiology	0.06	1.07	0.90–1.27	0.4649
General medicine vs Cardiology	–0.03	0.97	0.73–1.30	0.8352
**Professional title:**				
Professor vs attending physician	–0.06	0.95	0.83–1.08	0.4092
Associate professor vs attending physician	–0.11	0.90	0.80–1.01	0.0755
Resident vs attending physician	–005	1.05	0.92–1.20	0.4454
**Region:**				
North vs Northeast	–1.14	0.32	0.27–0.38	<0.0001
East vs Northeast	–0.71	0.49	0.42–0.58	<0.0001
Central vs Northeast	–0.16	0.85	0.73–0.99	0.0346
Southwest vs Northeast	–1.01	0.37	0.31–0.43	<0.0001
Northwest vs Northeast	–0.60	0.55	0.46–0.65	<0.0001

LDL–C – low density lipoprotein cholesterol, OR – odds ratio; CI – confidence interval, BMI – body mass index

DISCUSSION

The results of our study are consistent with previously published reports, which found that more than 60% of very high–risk patients and over 45% of high–risk patients did not attain their LDL–C goals, according to the 2007 Chinese lipid management guidelines, after at least 3 months of lipid–lowering treatment [10]. We found that physicians' knowledge of guideline–recommended LDL–C target goals significantly correlated with patients' LDL–C target goal achievement in China. Physician's knowledge varied depending on the hospital level, medical specialty, professional status, and geographic region. Working in a tier 1 or tier 2 hospital, being a resident physician, and specializing in neurology, endocrinology, or internal medicine were independent risk factors for lower LDL–C target goal achievement. Our results indicate that physician knowledge and characteristics affect the quality of lipid management in China, and more attention should be paid to this fact.

Previous studies have suggested that both patient– and physician–dependent factors contributed to LDL–C goal achievement failure [20–22]. Since the start of physician–initiated LDL–C treatment, more attention has been paid to studying the role of physicians in LDL–C goal achievement. Behavior change theory [23] suggests that knowledge should be the most important factor for behavior change. According to a global survey of physicians' perceptions of serum cholesterol management in 10 countries, 80.0% of

Table 5. Multivariate logistic regression analysis of LDL–C goal achievement rates

	Parameter estimation value	OR	95% CI	P–value
**Comorbidity (yes vs no):**				
Diabetes	1.08	2.94	2.75–3.14	1.0783
Coronary artery disease	0.61	1.84	1.72–1.97	0.6109
Cerebrovascular disease	0.29	1.33	1.22–1.44	0.2850
Peripheral arterial disease	–0.09	0.92	0.70–1.21	–0.0857
**Risk factors:**				
Hypertension (yes vs no)	0.01	1.01	0.95–1.08	0.7462
BMI >28kg/m^2	0.19	1.21	1.11–1.31	<0.0001
Male ≥45 years or Female ≥55 years (yes vs no)	0.14	1.15	1.03–1.29	0.0104
Smoking (yes vs no)	0.01	1.01	0.93–1.11	0.8020
Family history of early onset of ischemic cardiovascular disease (yes vs no)	0.04	1.04	0.94–1.15	0.4729
**Hospital level:**				
Tier 1 vs Tier3	0.44	1.56	1.34–1.81	0.4427
Tier 2 vs Tier3	0.15	1.16	1.06–1.27	0.1500
**Specialty:**				
Neurology vs Cardiology	0.45	1.57	1.40– 1.77	<0.0001
Endocrinology vs Cardiology	0.49	1.63	1.47–1.82	<0.0001
Geriatrics vs Cardiology	0.30	1.35	1.20–1.51	<0.0001
Internal Medicine vs Cardiology	0.46	1.58	1.39–1.80	<0.0001
General Medicine vs Cardiology	–0.09	0.91	0.75–1.11	0.3513
**Professional title:**				
Professor vs attending physician	–0.09	0.92	0.84–1.00	0.0420
Associate professor vs attending physician	–0.01	0.92	0.92–1.08	0.9055
Resident vs attending physician	0.09	1.00	1.00–1.19	0.0329
**Region:**				
North vs Northeast	–0.86	1.10	0.38–0.47	<0.0001
East vs Northeast	–0.85	0.42	0.39–0.48	<0.0001
Central vs Northeast	–0.69	0.43	0.45–0.56	<0.0001
Southwest vs Northeast	–0.89	0.50	0.37–0.46	<0.0001
Northwest vs Northeast	–0.87	0.41	0.38–0.47	<0.0001

LDL–C – low density lipoprotein cholesterol, , OR – odds ratio; CI – confidence interval, BMI – body mass index

physicians were concordant with dyslipidemia–management guidelines with regard to setting LDL–C goals for their patients, and 61.0% of them believed that a sufficient number of their patients attained their serum cholesterol goals. However, only 47.0% of their patients reached and maintained their serum cholesterol goals, and physicians expressed their frustration regarding their inability to effectively treat some CVD patients [24]. Another recent study in Croatia reported that 80.6% of physicians believed that they provided effective treatment to their dyslipidemia patients, but only 53.3% could accurately state the target LDL–cholesterol values for high–risk patients without consulting the most recent guidelines [25]. These reports suggest that physicians' knowledge of LDL–C target goals is an important factor for patients' LDL–C goal achievement.

In the current study, by evaluating correlations between physicians' knowledge of guideline–recommended LDL–C levels and LDL–C goal achievement, we aimed to investigate the physician's role in failing to attain recommended LDL–C goals in Chinese patients who were treated with lipid–lowering medications. We found that 74.5% of Chinese physicians recognized the importance of considering LDL–C serum concentration for treating dyslipidemia, and set target LDL–C goals that were concordant with the 2007 Chinese guidelines for 83.4% of their patients. Further analysis showed that 55.6% of these patients attained their serum LDL–C goal, indicating a success rate of 66.7%. We also found that the LDL–C goal achievement rate was significantly higher for patients whose physicians' knowledge of the LDL–C target goals was consistent with guideline recommendations, compared with those whose physicians' knowledge was inconsistent with the guidelines (60.4% vs 31.1%, $P<0.0001$). This finding supports the idea that enhancing Chinese physicians' awareness of LDL–C target goals may improve the quality of lipid control in China. We also found that hospital level, as well as medical specialty and professional status were significantly related to physicians' knowledge and their patients' LDL–C goal achievement. Physicians from tier 1 and tier 2 hospitals, who were specializing in endocrinology, internal medicine, or general medicine,

and resident physicians demonstrated lower awareness of guideline–recommended LDL–C target goals than their colleagues. Multivariate logistic regression analysis revealed a relatively strong correlation between physician characteristics and physician–suggested LDL–C target goals and guideline–recommended LDL–C goals. These findings suggest that certain physician characteristics and inadequate knowledge of guideline–recommended LDL–C goals may be important reasons for patients' failure to attain their LDL–C target goals in China. Guideline–recommended LDL–C goals should be considered among the most important pieces of information delivered to physicians. These results also suggest that continuing medical education strategy in China should be based on physician characteristic.

This is the first study to investigate factors underlying suboptimal LDL–C goal achievement rates in China from the physicians' perspective in an innovative attempt to improve the quality of cardiovascular disease prevention in China. There are, however, certain limitations to our study. Our results point to inadequate awareness among Chinese physicians regarding lipid control. However, the questionnaire completed by physicians addressed their knowledge of LDL–C only. Thus, our results do not provide a comprehensive view of all aspects of the physicians' skills regarding lipid management. Furthermore, due to our focus on physicians' characteristics and lipid knowledge in our study, we did not collect data on all potential confounding factors, such as the physician' age, gender, and behavior regarding lipid management, as well as patients' lifestyle habits that might have influenced LDL–C goal achievement. However, there are some advantages to our study. First, this is a large–scale cohort study, which included patients' and physicians' medical information together, related to lipid medication; therefore, we can directly analyze the relationship between physicians' knowledge and patients' LDL–C goal achievement. Second, the physicians' information in our cohort was collected with the randomized cohort stratified sample method, so it is representative of physician characteristics throughout China. Physician characteristics included their hospital level, specialty, professional status, and service regions, so we can clearly tell the effect of physician characteristics on knowledge of guideline–recommended LDL–C target goals and on LDL–C goal achievement. Thus, we were able to uncover shortcomings in China regarding physician characteristics and knowledge. Although we chose only 2 questions, which focused on LDL–C, those 2 questions are fundamental to lipid control relating to physician behavior. In this study, we used 2007 Chinese guideline–recommended LDL–C target goals and did not use the updated 2013 ESC guidelines. The reason for this was that LDL–C target goals in the Chinese guidelines were the most widely disseminated in China. This way we could tell whether or not the effect of knowledge of LDL–C target goals on LDL–C target achievement related to physician characteristics.

Our study has identified some shortcomings in health services in Chinese community medical centers (tier 1 hospitals) regarding physician characteristics and inadequate knowledge. The lower percentage of specialists and professors in community medical centers result in less knowledge and lower LDL–C target achievement. This may be just the tip of the iceberg for medical service shortcomings in China. Since community hospitals have been considered as the first bastion of chronic disease prevention in China, efficient work by community physicians is of great importance for the health of the population [19]. It is imperative to improve the level of health services in community medical centers. Arduous educational efforts must be ongoing in Chinese community medical centers, and more attention should be paid to physicians' knowledge of LDL–C target goals.

CONCLUSIONS

The results of our study suggest that LDL–C goal achievement in dyslipidemia patients in China is associated with physicians' characteristics and their knowledge of LDL–C guidelines. Deficiencies in physicians' knowledge of LDL–C targets reveal serious shortcomings in health services in China. Strategies aimed at enhancing familiarity with and acceptance of LDL-C guidelines by Chinese physicians based on the region, professional status, medical specialty, and hospital level may be an efficient way to improve both guideline adherence and LDL–C goal achievement.

PAPERS

Acknowledgments: We sincerely thank Dr Philippe Brudi for his great contribution to the global DYSIS study protocol design.

Ethics approval: Our study protocol was approved by the ethics committee of each participating hospital

Funding: This study was funded by a research grant from Merck & Co., Ltd (IISP#39298).

Authorship contributions: Dr Hu Dayi conceived and initiated the study, supervised its conduct and data analysis and had primary responsibility for writing the report. Dr Ding Rongjing wrote the report of this study. Dr Ding Rongjing, Dr Ye Ping, Zhao Shuiping, Dr Zhao Dong, and Dr Yan Xiaowei contributed to the conception, design, and assembly of all data. All of the authors reviewed the report and provided critical input for its revision.

Competing interests: The authors have completed the Unified Competing Interest form at www.icmje.org/coi_disclosure.pdf (available on request from the corresponding author) and declare no conflicts of interests.

REFERENCES

1 Expert Panel on Detection E. Treatment of High Blood Cholesterol in A. Executive Summary of The Third Report of The National Cholesterol Education Program (NCEP) Expert Panel on Detection, Evaluation, And Treatment of High Blood Cholesterol In Adults (Adult Treatment Panel III). JAMA. 2001;285:2486-97. Medline:11368702 doi:10.1001/jama.285.19.2486

2 Graham I, Atar D, Borch-Johnsen K, Boysen G, Burell G, Cifkova R, et al. European guidelines on cardiovascular disease prevention in clinical practice: executive summary. Fourth Joint Task Force of the European Society of Cardiology and other societies on cardiovascular disease prevention in clinical practice (constituted by representatives of nine societies and by invited experts). Eur J Cardiovasc Prev Rehabil. 2007;14 Suppl 2:E1-40. Medline:17726406 doi:10.1097/01.hjr.0000277984.31558.c4

3 Grundy SM, Cleeman JI, Merz CN, Brewer HB Jr, Clark LT, Hunninghake DB, et al. Implications of recent clinical trials for the National Cholesterol Education Program Adult Treatment Panel III guidelines. Circulation. 2004;110:227-39. Medline:15249516 doi:10.1161/01.CIR.0000133317.49796.0E

4 National Cholesterol Education Program Expert Panel on Detection E. Treatment of High Blood Cholesterol in A. Third Report of the National Cholesterol Education Program (NCEP) Expert Panel on Detection, Evaluation, and Treatment of High Blood Cholesterol in Adults (Adult Treatment Panel III) final report. Circulation. 2002;106:3143-421. Medline:12485966

5 Wilson PW, D'Agostino RB, Levy D, Belanger AM, Silbershatz H, Kannel WB. Prediction of coronary heart disease using risk factor categories. Circulation. 1998;97:1837-47. Medline:9603539 doi:10.1161/01.CIR.97.18.1837

6 Sager HB, Linsel-Nitschke P, Mayer B, Lieb W, Franzel B, Elsasser U, et al. Physicians' perception of guideline-recommended low-density lipoprotein target values: characteristics of misclassified patients. Eur Heart J. 2010;31:1266-73. Medline:20219745 doi:10.1093/eurheartj/ehq026

7 Wägner AM, Perez A, Calvo F, Bonet R, Castellvi A, Ordonez J. Apolipoprotein(B) identifies dyslipidemic phenotypes associated with cardiovascular risk in normocholesterolemic type 2 diabetic patients. Diabetes Care. 1999;22:812-7. Medline:10332687 doi:10.2337/diacare.22.5.812

8 Baigent C, Keech A, Kearney PM, Blackwell L, Buck G, Pollicino C, et al. Efficacy and safety of cholesterol-lowering treatment: prospective meta-analysis of data from 90,056 participants in 14 randomised trials of statins. Lancet. 2005;366:1267-78. Medline:16214597 doi:10.1016/S0140-6736(05)67394-1

9 Cholesterol Treatment Trialists C, Kearney PM, Blackwell L, Collins R, Keech A, Simes J, et al. Efficacy of cholesterol-lowering therapy in 18,686 people with diabetes in 14 randomised trials of statins: a meta-analysis. Lancet. 2008;371:117-25. Medline:18191683 doi:10.1016/S0140-6736(08)60104-X

10 Zhao S, Wang Y, Mu Y, Yu B, Ye P, Yan X, et al. Prevalence of dyslipidaemia in patients treated with lipid-lowering agents in China: results of the DYSlipidemia International Study (DYSIS). Atherosclerosis. 2014;235:463-9. Medline:24950001 doi:10.1016/j.atherosclerosis.2014.05.916

11 Wu YF. The second multi-center survey of dyslipidemia management in China: goal attainment rate and related factors. Zhonghua Xin Xue Guan Bing Za Zhi. 2007;35:420-7. Medline:17711683

12 Gitt AK, Lautsch D, Ferrieres J, Kastelein J, Drexel H, Horack M, et al. Low-density lipoprotein cholesterol in a global cohort of 57,885 statin-treated patients. Atherosclerosis. 2016;255:200-9. Medline:27667299 doi:10.1016/j.atherosclerosis.2016.09.004

13 How to attain the ambitious goals for health reform in China. Lancet. 2015;386:1419. Medline:26466023 doi:10.1016/S0140-6736(15)00452-3

14 Yarzebski J, Bujor CF, Goldberg RJ, Spencer F, Lessard D, Gore JM. A community-wide survey of physician practices and attitudes toward cholesterol management in patients with recent acute myocardial infarction. Arch Intern Med. 2002;162:797-804. Medline:11926854 doi:10.1001/archinte.162.7.797

15 Cacoub P, Tocque-Le Gousse E, Fabry C, Hermant S, Petzold L. Application in general practice of treatment guidelines for patients with dyslipidaemia: the RESPECT study. Arch Cardiovasc Dis. 2008;101:715-21. Medline:19059566 doi:10.1016/j.acvd.2008.09.011

PAPERS

REFERENCES

16 Davidson MH, Maki KC, Pearson TA, Pasternak RC, Deedwania PC, McKenney JM, et al. Results of the National Cholesterol Education (NCEP) Program Evaluation ProjecT Utilizing Novel E-Technology (NEPTUNE) II survey and implications for treatment under the recent NCEP Writing Group recommendations. Am J Cardiol. 2005;96:556-63. Medline:16098311 doi:10.1016/j.amjcard.2005.04.019

17 Frolkis JP, Pearce GL, Nambi V, Minor S, Sprecher DL. Statins do not meet expectations for lowering low-density lipoprotein cholesterol levels when used in clinical practice. Am J Med. 2002;113:625-9. Medline:12505111 doi:10.1016/S0002-9343(02)01303-7

18 Hwang JY, Jung CH, Lee WJ, Park CY, Kim SR, Yoon KH, et al. Low Density Lipoprotein Cholesterol Target Goal Attainment Rate and Physician Perceptions about Target Goal Achievement in Korean Patients with Diabetes. Diabetes Metab J. 2011;35:628-36. Medline:22247906 doi:10.4093/dmj.2011.35.6.628

19 Guan F, Xie J, Wang GL, Wang JH, Wang JS, Yu JM, et al. Community-wide survey of physicians' knowledge of cholesterol management. Chin Med J (Engl). 2010;123:884-9. Medline:20497682

20 Casula M, Tragni E, Catapano AL. Adherence to lipid-lowering treatment: the patient perspective. Patient Prefer Adherence. 2012;6:805-14. Medline:23152673

21 Plana N, Ibarretxe D, Cabre A, Ruiz E, Masana L. Prevalence of atherogenic dyslipidemia in primary care patients at moderate-very high risk of cardiovascular disease. Cardiovascular risk perception. Clin Investig Arterioscler. 2014;26:274-84. Medline:24931442 doi:10.1016/j.arteri.2014.04.002

22 McCrate F, Godwin M, Murphy L. Attainment of Canadian Diabetes Association recommended targets in patients with type 2 diabetes: a study of primary care practices in St John's, Nfld. Can Fam Physician. 2010;56:e13-9. Medline:20090056

23 Cabana MD, Rand CS, Powe NR, Wu AW, Wilson MH, Abboud PA, et al. Why don't physicians follow clinical practice guidelines? A framework for improvement. JAMA. 1999;282:1458-65. Medline:10535437 doi:10.1001/jama.282.15.1458

24 Erhardt LR, Hobbs FD. A global survey of physicians' perceptions on cholesterol management: the From The Heart study. Int J Clin Pract. 2007;61:1078-85. Medline:17577295 doi:10.1111/j.1742-1241.2007.01420.x

25 Reiner Z, Sonicki Z, Tedeschi-Reiner E. Physicians' perception, knowledge and awareness of cardiovascular risk factors and adherence to prevention guidelines: the PERCRO-DOC survey. Atherosclerosis. 2010;213:598-603. Medline:20947087 doi:10.1016/j.atherosclerosis.2010.09.014
